# Sequential and Coordinated Actions of c-Myc and N-Myc Control Appendicular Skeletal Development

**DOI:** 10.1371/journal.pone.0018795

**Published:** 2011-04-11

**Authors:** Zi-Qiang Zhou, Chia-Yi Shung, Sara Ota, Haruhiko Akiyama, Douglas R. Keene, Peter J. Hurlin

**Affiliations:** 1 Shriners Hospitals for Children Portland, Portland, Oregon, United States of America; 2 Department of Cell and Developmental Biology, Oregon Health and Science University, Portland, Oregon, United States of America; 3 Department of Orthopaedics, Kyoto University, Kyoto, Japan; University of Western Ontario, Canada

## Abstract

**Background:**

During limb development, chondrocytes and osteoblasts emerge from condensations of limb bud mesenchyme. These cells then proliferate and differentiate in separate but adjacent compartments and function cooperatively to promote bone growth through the process of endochondral ossification. While many aspects of limb skeletal formation are understood, little is known about the mechanisms that link the development of undifferentiated limb bud mesenchyme with formation of the precartilaginous condensation and subsequent proliferative expansion of chondrocyte and osteoblast lineages. The aim of this study was to gain insight into these processes by examining the roles of c-Myc and N-Myc in morphogenesis of the limb skeleton.

**Methodology/Principal Findings:**

To investigate c-Myc function in skeletal development, we characterized mice in which floxed c-Myc alleles were deleted in undifferentiated limb bud mesenchyme with *Prx1-Cre*, in chondro-osteoprogenitors with *Sox9-Cre* and in osteoblasts with *Osx1-Cre*. We show that c-Myc promotes the proliferative expansion of both chondrocytes and osteoblasts and as a consequence controls the process of endochondral growth and ossification and determines bone size. The control of proliferation by c-Myc was related to its effects on global gene transcription, as phosphorylation of the C-Terminal Domain (pCTD) of RNA Polymerase II, a marker of general transcription initiation, was tightly coupled to cell proliferation of growth plate chondrocytes where c-Myc is expressed and severely downregulated in the absence of c-Myc. Finally, we show that combined deletion of *N-Myc* and *c-Myc* in early limb bud mesenchyme gives rise to a severely hypoplastic limb skeleton that exhibits features characteristic of individual *c-Myc* and *N-Myc* mutants.

**Conclusions/Significance:**

Our results show that N-Myc and c-Myc act sequentially during limb development to coordinate the expansion of key progenitor populations responsible for forming the limb skeleton.

## Introduction

The limb skeleton develops from limb buds that are initially composed of rapidly proliferating multipotent mesenchymal stem cells encased in ectoderm. These cells are maintained in a proliferative and undifferentiated state primarily by the secretion of Fgf and Wnt ligands from ectoderm [1], [2]. As the limb bud expands, centrally located cells begin to condense and their increasing distance from surface ectoderm-derived proliferative signals corresponds to their exit from the cell cycle [3]. From condensing mesenchyme emerge progenitors of chondrogenic, osteogenic, tendon, ligament and other connective tissues lineages [4], [5], [6], [3]. Further outgrowth of the limb skeleton is governed largely by the coordinated homeostatic expansion and differentiation of chondrocytes within the longitudinally oriented structure known as the growth plate, and osteoblasts in immediately adjacent perichondrial tissue [7], [8]. During bone growth a subset of slowly proliferating chondrocyte progenitors within the growth plate continuously enter into a program of terminal differentiation, where they first undergo proliferative expansion and then undergo hypertrophy and die. The extracellular matrix laid down by terminally differentiated chondrocytes serves as a template for invasion of blood vessels, which in turn serve as a conduit for the emigration of osteoblasts from their perichondrial residence onto the cartilaginous template. The dying chondrocytes and invading osteoblasts establish the ossification front where differentiating osteoblasts then promote bone mineralization and ossification in the process of endochondral ossification.

Chondrogenic and osteogenic lineages arising from condensing mesenchyme in the limb bud are defined by the expression and activity of the transcription factors Sox9 and Runx2/Cbfa1 respectively [8]. Sox9 regulates a variety of genes encoding extracellular matrix components that guide chondrocyte behavior and function, and its deletion prior to condensation in the limb bud completely blocks cartilage formation [9], [10]. In contrast to *Sox9*, deletion of *Runx2* completely blocks development of the osteoblast lineage and therefore bone ossification [11], [12], [13]. *Sox9* deletion in the early limb bud also blocks *Runx2* expression and osteogenesis because the osteogenic lineage is derived from condensing mesenchyme of the limb bud that fails to properly form in the absence of *Sox9* [10]. Furthermore, Sox9 controls this early step in osteochondro-progenitor cell fate determination by directly interacting with Runx2 and inhibiting *Runx2* transcriptional activity and its ability to promote osteogenesis [14].

Runx2 is not only a cell autonomous regulator of the osteogenic lineage, but also has cell non-autonomous effects on osteoblasts by promoting chondrocyte maturation and expression of Vegfa in prehypertrophic chondrocytes [15], [16]. Vegfa produced in the prehypertrophic chondrocytes in turn acts on endothelial cells outside the cartilage to direct vascular recruitment and invasion into the matrix formed by terminally differentiating chondrocytes at the ossification front [16], [17], [18], [19], [20]. Like *Runx2*, *Indian Hedgehog* (*Ihh*) expression is also initiated in prehypertrophic chondrocytes and Ihh appears to have a positive influence on blood vessel development or function [21], [22]. Ihh secreted from prehypertrophic chondrocytes also has a more well-characterized role in governing proliferation of chondrocytes in the growth plate, as well as osteoblasts in the perichondrium, by binding to its receptor Patched-1 (Ptch-1) at these locations and by stimulating production of Parathyroid hormone-related peptide (PTHrP) [8]. Thus, chondrocyte maturation directed by Runx2 and IHH indirectly influences osteoblast proliferation to promote endochondral ossification.

Despite major advances in the understanding of chondrocyte and osteoblast lineage determination and endochondral growth, little is known about the mechanisms that link the development of undifferentiated limb bud mesenchyme with formation of the precartilaginous condensation and subsequent development and expansion of chondrocyte and osteoblast lineages. Potential candidates for coordinating these activities are Myc family transcription factors, particularly c-Myc and N-Myc, which are known to strongly influence the development and maintenance of pluripotent and multipotent in a number of homeostatic tissues (reviewed in [23], [24]). *N-Myc* and *c-Myc* are sequentially expressed in developing limbs, with *N-Myc* expressed primarily in undifferentiated limb bud mesenchyme and *c-Myc* expressed in proliferating chondrocytes of the growth plate and osteoblasts in the perichondrium (see below). N-Myc is known to promote the proliferative expansion of undifferentiated limb bud mesenchyme, which in turn influences the formation of condensing mesenchyme and production of osteochondro-progenitors [25], [3]. In contrast, c-Myc has been implicated in the control of chondrocyte proliferation and differentiation in the growth plate [26], [27], [28], [29], but its effects on cartilage formation and endochondral ossification are poorly defined. Using conditional deletion mutants, we show here that c-Myc plays an important role in endochondral ossification and bone growth by promoting efficient proliferative expansion of both chondrocyte and osteoblast lineages. Consistent with N-Myc and c-Myc playing unique and complementary roles in limb skeletal development, combined deletion of *N-Myc* and *c-Myc* in the early limb bud gives rise to a severe limb miniaturization phenotype that exhibits features characteristic of individual *c-Myc* and *N-Myc* mutants. Together, our results indicate that N-Myc and c-Myc function to maintain and expand distinct and sequentially formed progenitor populations responsible for forming the limb skeleton.

## Results

### c-Myc does not influence limb bud outgrowth, but controls the initial growth of the limb skeleton

**Figure 1:**
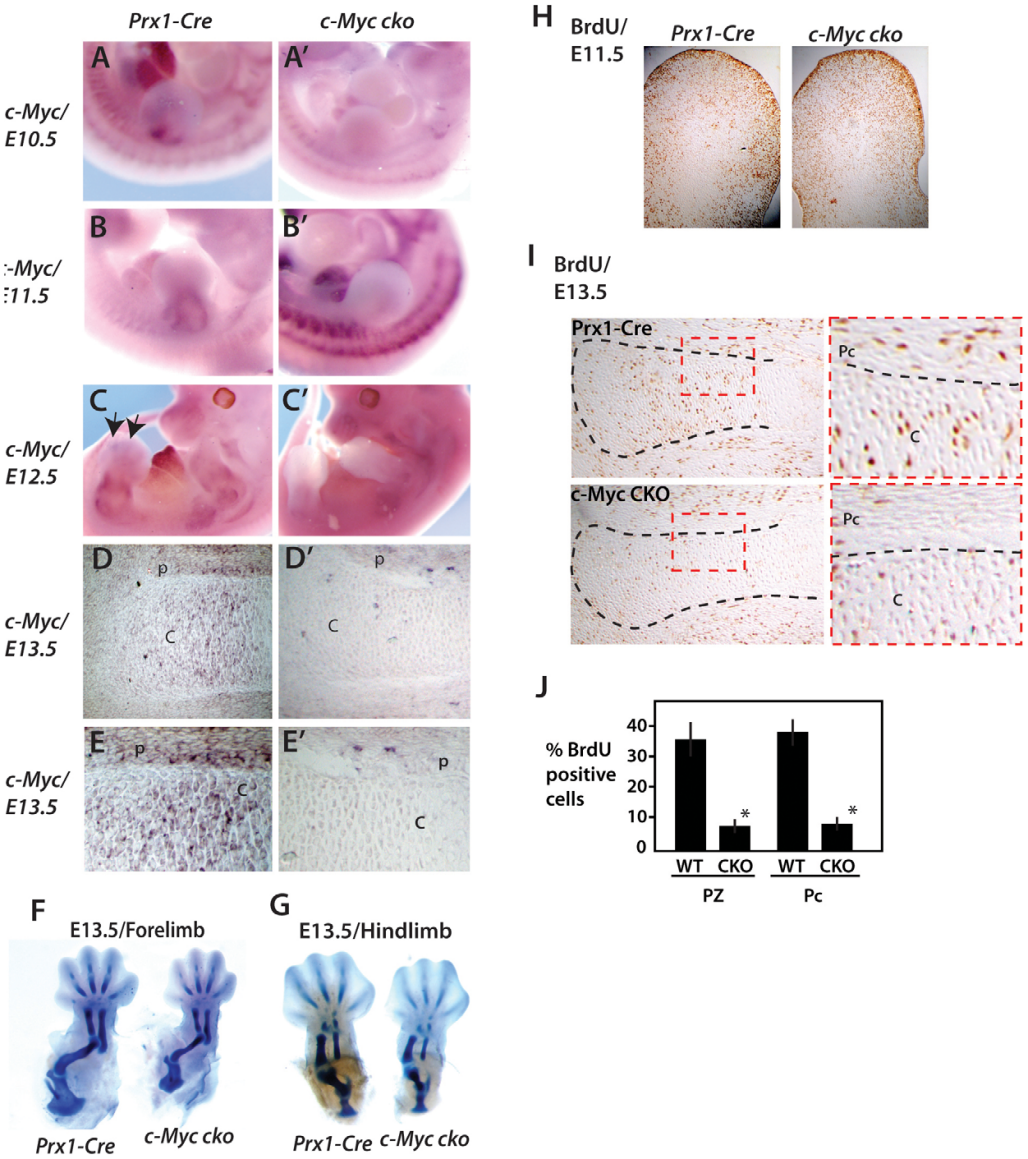
c-Myc plays an early role in appendicular skeletal development. (A–C') Whole mount in situ hybridization of *c-Myc* in forelimb buds at E10.5, E11.5 and E12.5 of wildtype and Prx1-Cre c-Myc mutant embryos. (D, D') In situ hybridization for *c-Myc* in sections of proximal tibia at E13.5 of wildtype and Prx1-Cre c-Myc mutant embryos. (E, E') Higher magnification views of proximal tibia sections shown in D and D'. Cartilage and (C) and perichondrium (P) are indicated. (H, I) BrdU staining in limb buds at E11.5 and proximal tibia at E13.5 respectively. (J) Summary of BrdU-positive cells in the proliferative zone (PZ) and perichondrium (Pc).

**Figure 2:**
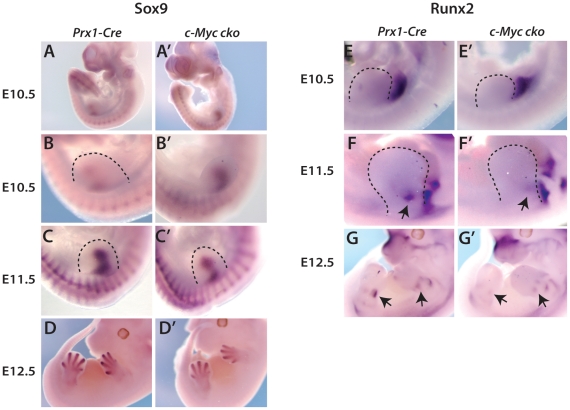
Limb bud deletion of *c-Myc* affects *Sox9* and *Runx2* expression and formation of cartilage anlage. Analysis of *Sox9* expression (A-D') and Runx2 expression (E-G') by in situ hybridization in forelimbs of *Prx1-Cre* control and *c-Myc* cko embryos at the indicated stages. Dashed lines delineate the limb bud. (H, H') Alcian blue staining of forelimb and hindlinb cartilage anlage of control and c-Myc cko embryos at E13.5.

To investigate the role of *c-Myc* in limb development, *c-Myc* conditional alleles [30] were deleted with *Prx1-Cre*, which is active in undifferentiated limb bud mesenchyme prior to formation of Sox9-dependent cartilage [31], [10]. At E10.5, *c-Myc* is expressed at the base of the forelimb bud (Fig. 1A) in a region near to where Sox9 is expressed (see Fig. 2A–B'), but not in undifferentiated limb bud mesenchyme. At E11.5 *c-Myc* is expressed most intensely in a ring of tissue in the central/proximal limb bud (Fig. 1B) that appears to surround and perhaps partly overlap with the expression domains of both Sox9 and Runx2 (Fig. 2C and Fig. 2G respectively). At E12.5, in addition to a ring pattern of expression in the more proximal limb region (Fig. 1C) that overlaps Runx2 expression (Fig. 2H), *c-Myc* is visible in the initial cartilage templates of the autopod (Fig. 1C). At E13.5 *c-Myc* is expressed in both chondrocytes of cartilage anlage and perichondrium where osteoblasts reside (Fig. 1C). Consistent with early and robust *Prx1-Cre* activity in limb bud mesenchyme that precedes Sox9 expression [5], [31], *c-Myc* expression is extinguished in E10.5 and E12.5 limb buds of *Prx1-Cre c-Myc* ^fl/fl^ (*c-Myc* cko) mice and in chondrocyctes and perichondrial cells at E13.5 (Fig. 1A', B', C'). The residual proximal limb bud *c-Myc* expression observed in *c-Myc* cko limb buds at E12.5 is likely in non-targeted muscle cells streaming into the limb buds since *c-Myc* expression is apparent in muscle cells at later stages (not shown).

Consistent with *c-Myc* playing a role in development of the initial cartilage template of the limb skeleton, Alcian blue staining at E13.5 revealed a reduction in the size of all limb skeletal elements (Fig. 1F,G), including the elements of the shoulder (scapula) and hindlimb girdle (ilium and ishium), which do not develop from mesenchyme within the limb bud proper [31]. The scapula, which develops from the somatopleure of the forelimb field (cranial part of the scapula that articulates with the humerus) and somitic dermomytome (scapula blade) [31], was more affected than the illium/ishium, a result consistent with *Prx1-Cre* being more active in the emerging forelimb bud and surrounding limb field than comparable regions of the developing hindlimb bud [10], [32].

To examine the relationship between the decreased size of the cartilaginous elements and cell proliferation, BrdU incorporation assays performed at E11.5 and E13.5 (Fig. 1H, I). Whereas no consistent change in the pattern of BrdU incorporation was evident at E11.5, there was a strong reduction in BrdU incorporation in both chondrocytes of the emerging growth plate and in perichondrial cells at E13.5 (Fig. 1H, I, J).

### *Sox9* and *Runx2* expression in the developing limb skeleton are disrupted by c-Myc-deficiency

To further evaluate how *c-Myc* deletion influenced chondrocyte and osteoblast development in the developing limb, *Sox9* and *Runx2* expression was examined. At E10.5, *c-Myc* mutants consistently showed upregulation and/or expanded expression of *Sox9* (Fig. 2A–B' also see Fig. 7IV–J), which marks the precartilaginous condensation. *Sox9* expression was similarly upregulated/expanded in hindlimb buds at E11.5 (not shown), a developmental stage roughly equivalent to E10.5 in forelimb buds. In contrast, mutant forelimb buds at E11.5 consistently exhibited reduced intensity and area of *Sox9* expression compared to *Prx1-Cre* controls (Fig. 2C, C'). *Sox9* expression at E12.5, now marking the cartilage template of the developing limb skeleton, remained weak in *c-Myc* mutants (Fig. 2D, D').

*Runx2*, encoding the master regulator of the osteoblast lineage, was expressed in lateral plate mesoderm adjacent to the emerging forelimb bud at E10.5 as previously described [5], and Runx2 expression in this region appeared to not be appreciably altered by *Prx1-Cre* deletion of *c-Myc* (Fig. 2G, G'). At E11.5 and E12.5, the initial *Runx2* expression domains in forelimb bud mesenchyme (Fig. 2H–I'), which partially overlap *Sox9* expression (Fig. 2C, D, also see [5], were reduced in *c-Myc* mutants. These results suggest that reduced skeletal size caused by loss of c-Myc is related to inefficient expansion of the chondrocyte and osteoblast lineages.

### Control of endochondral bone growth and ossification by *c-Myc*

**Figure 3:**
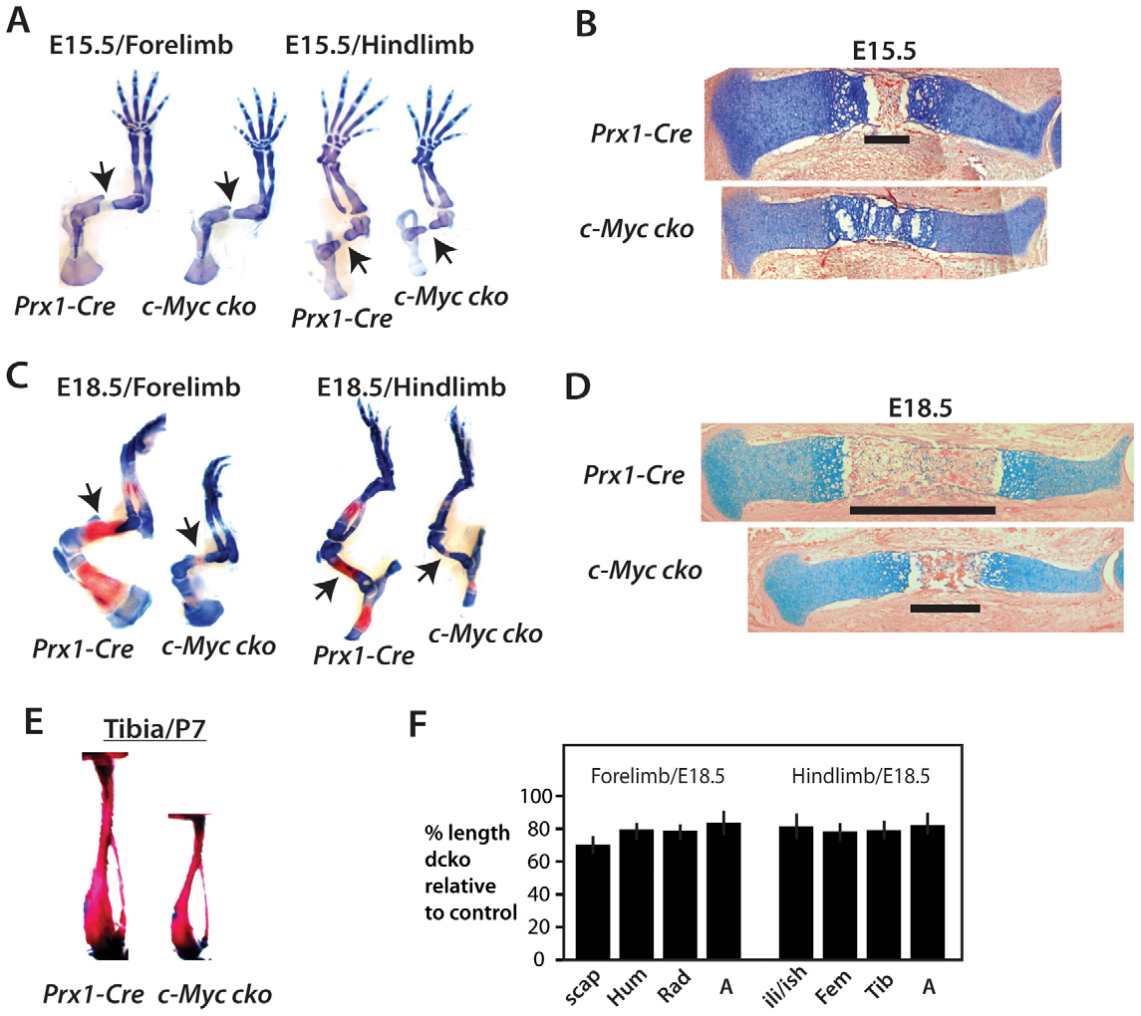
Delayed endochondral ossification and suppressed bone growth caused by *c-Myc* deficiency. Alcian blue and Alizarin red stained forelimb and hindlimb skeletal preparations at E15.5 (A) and E18.5 (C). Arrows point to primary ossification centers of the humerus and femur to indicate delayed (E15.5) and smaller (E18.5) ossification centers in *c-Myc* deficient limbs. (B, D) Alcian blue and hemotoxylin and eosin (H&E) stained sections at E15.5 and E16.5. Bars indicate length of primary ossification center. (E) Alizarin red stained tibia at P7 showing small, but fully ossified bone in c-Myc mutants. (F) Measurement of bone length at E18.5. Autopod length was from the tip of digit 3 to the base of the lunate carpal bone.

At E15.5 the coordinated actions of chondrocytes within the growth plate and osteoblasts in the perichondrium initiate the process of endochondral ossification. Alcian blue staining of skeletons at E15.5 and E18.5 showed *c-Myc* mutant elements continued to be reduced in size and exhibited small primary ossification centers (Fig. 3A–D), consistent with delayed and/or defective endochondral ossification. von Kossa staining (mineralized bone) at E15.5 confirmed a delay in forming the primary ossification center (not shown) At postnatal day 7, tibias were small but fully ossified (Fig. 3E). Measurements of bone length at E18.5 showed a similar reduction in the size of all appendicular elements, with the scapula most affected (Fig. 3F).

### The activities of c-Myc in limb development are limited to after formation of chondro-osteoprogenitors

Although c-Myc appears to not be expressed in undifferentiated limb bud mesenchyme (Fig. 1A, B), to rule out that the effects on Sox9 and Runx2 expression and the small skeleton produced by *Prx1-Cre* deletion of *c-Myc* might be due to cryptic c-Myc activity in early undifferentiated limb bud mesenchyme, we deleted *c-Myc* with Sox9-Cre, which is not active prior to development of chondro-osteoprogenitors and the precartilaginous condensation [5]. Sox9-Cre deletion of *c-Myc* resulted in small limb skeletal elements with delayed and small primary ossification centers (Fig. S1), a phenotype essentially identical to that caused by *Prx1-Cre* deletion. These results indicate that the function of c-Myc in appendicular skeletal development is largely, if not wholly, restricted to after formation of chondro-osteoprogenitors.

In addition, deletion of *c-Myc* with *Osx1-Cre*, which is active in osteoblasts after Runx2-dependent commitment to the osteoblast lineage [33], had no apparent effect on endochondral ossification or bone growth (Fig. S2). The latter results suggest that either c-Myc functions at a relatively early step(s) in osteoblast development that is prior to the role Osterix plays in osteoblast differentiation [34] or that it does not directly regulate osteoblasts (see Discussion).

### *c-Myc* controls the number of differentiated chondrocytes produced in the growth plate

**Figure 4:**
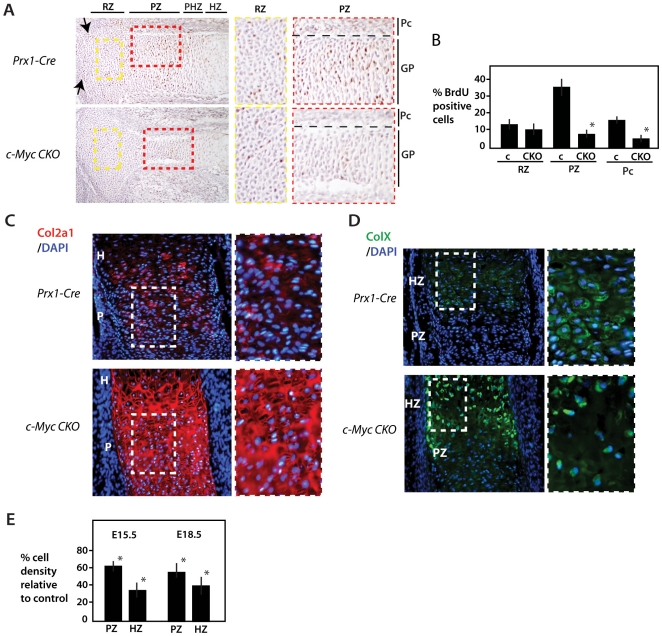
Control of cell proliferation and cell density in the growth plate by c-Myc. (A) BrdU labeling of control and *Prx1-Cre c-Myc* mutant proximal tibia at E16.5. Approximate locations of specific regions of the growth plate (GP) and perichondrium (Pc) are indicated and higher magnification images for the RZ (yellow dashed box) and PZ (red dashed box) are shown at right. The division between cartilage and perichondrium in (A) is marked by the black dashed line. Arrow point to articular regions that show reduced proliferation in *c-Myc* mutants. (B) Percentage of BrdU labeled cells in comparable regions of the RZ, PZ and perichondrium (Pc) of *Prx1-Cre* control (c) tibias and *Prx1-Cre c-Myc* mutant tibias (see Materials and Methods for details). C) Proximal tibias of the indicated stains at E16.5 stained for Col2a1 and DAPI. Higher magnification views are at right. (D) Proximal tibias stained for ColX and DAPI with higher magnification views at right. (E) The percentage change in cell density (cells per unit area) of *c-Myc* mutants compared to controls in comparable regions of PZ and HZ as determined from sections stained with DAPI alone (see Materials and Methods). *p<0.01.

Differentiated chondrocytes play critical roles in endochondral ossification by producing factors such as Vegfa and IHH that signal to osteoblasts in adjacent perichondrium. Analysis of BrdU incorporation at E16.5, when endochondral ossification has commenced, revealed a continued diminution of proliferation (Fig. 4A, B). Very similar results were observed with *c-Myc* deficient tibias at E18.5 (not shown). In addition to decreased proliferation in the proliferative zone of the growth plate, there appeared to be lower levels of proliferation in the epiphyseal articulating surface (Fig. 4A, see arrows), suggesting that c-Myc may play a role in the development of articular cartilage.

The decreased chondrocyte proliferation in the growth plate corresponded to a significant decrease in the density of cells in the proliferative and hypertrophic zones of *c-Myc* deficient growth plates as determined by counting DAPI-positive cells (Fig. 4C, E). The reduced cell density caused by loss of *c-Myc* is associated with an increased abundance of Collagen Type 2a1 (Col2) in the extracellular matrix. An even greater decrease in cell density was observed in the hypertrophic region marked by Collagen Type X (ColX) (Fig. 4D, E). In contrast to the prehypertrophic and hypertrophic regions of the growth plate, the cell density and already low level of proliferation in the resting zone of proximal tibias was much less affected by loss of c-Myc (Fig. 4A and not shown). These results support a role for *c-Myc* in initiating and promoting the proliferative expansion of chondrocytes in the growth plate. The decrease in proliferation and in *Runx2* expression in the perichondrium also suggests that c-Myc regulates expansion of osteoblasts, but the less-well defined architecture of the perichondrium compared to the growth plate is not amenable to an accurate determination of how loss of c-Myc might affect cell density at this location.

### Defective endochondral ossification is linked to decreased *Vegfa* expression and disrupted vasculogenesis

**Figure 5:**
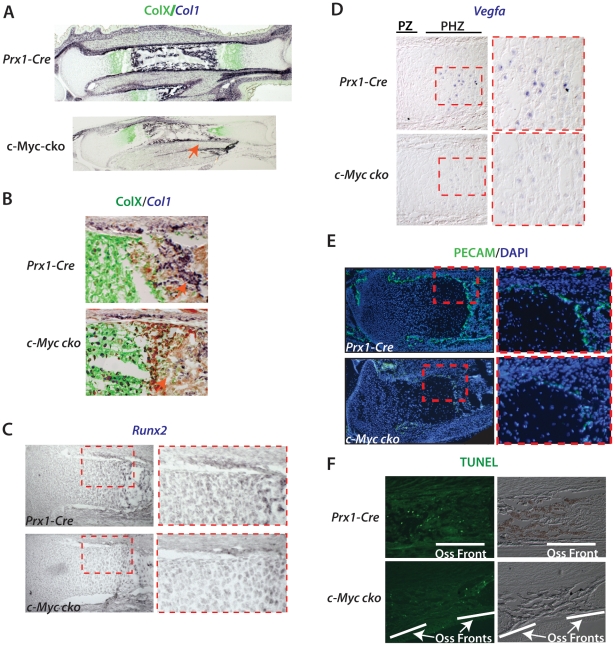
Disrupted endochondral ossification in *c-Myc* mutants is linked to decreased numbers of osteoblasts and poor vascularization. (A) Combined ColXa1 immunohistochemistry and *Col1* in situ hybridization at E18.5 showing a smaller hypertrophy zone (marked by ColXa1 – green) and reduced *Col1* (dark staining) in the perichondrium of *c-Myc* mutant tibia. (B) Higher magnification view of the primary ossification center and adjacent perichondrium showing fewer *Col1*-positive osteoblasts (orange arrow) and a thinner *Col1*-positive layer in the perichondrium of *c-Myc* mutant tibia. Red staining is pan-cytokeratin.(C) Runx2 in situ hybridization. (D) *Vegfa* in situ hybridization showing reduced expression in hypertrophic chondrocytes of *c-Myc* mutant tibia at E18.5. Approximate locations of proliferative and prehypertrophic zones are indicated and higher magnification images of boxed regions are shown at right. (E) PECAM immunohistochemistry indicating reduced vascularization of the primary ossification center and perichondrium of *c-Myc* deficient tibia. Red boxed area is shown at higher magnification on right. (F) TUNEL staining for apoptotic cells at the ossification fronts of control and *c-Myc* mutant tibia.

To further characterize endochondral growth and ossification in *c-Myc* mutants, we examined the relationship between ColX protein expression in hypertrophic chondrocytes of the growth plate and *Collagen type I* (*Col1*) mRNA expression in osteoblasts. In E18.5 tibias, the area of hypertrophic ColX expression was reduced, but what was more striking was a marked decrease in *Col1* expression in perichondrium and bone collar regions as well as in the primary ossification center (Fig. 5A, B). The osteoblast marker *Osteocalcin* was strongly reduced in the same regions *(not shown)*. In addition, *Runx2* expression in perichondrium was reduced at E18.5 (Fig. 5C), suggesting that osteoblastogenesis is compromised in *c-Myc* mutants.

*Runx2* expression was also reduced in the growth plate (Fig. 5C and also see Fig. 9IIG). Because *Vegfa* expression in the growth plate is dependent on *Runx2* [16], we examined *Vegfa* in control and mutant tibias. Both the number of cells expressing *Vegfa* in hypertrophic chondrocytes and the intensity of it's expression was reduced in the absence of *c-Myc* (Fig. 5D). Consistent with reduced expression of *Vegfa*, blood vessel formation, as marked by expression of PECAM (CD31) was markedly decreased in the perichondrium and ossification front of *c-Myc* mutant tibias (Fig. 5E), but not in adjacent non-skeletal tissues (data not shown). These results suggest that the decreased number of osteocytes within the primary ossification center and the delayed and defective endochondral ossification observed in *c-Myc* mutants may be partly due to defects in vascularization.

Another potential mechanism by which *c-Myc* might influence the number of differentiated chondrocytes in the growth plate and endochondral ossification is defective apoptosis. However, *c-Myc* deficiency caused no increase in apoptosis as measured by TUNEL staining and Lysotracker staining in the growth plate or perichondrium of tibias at E15.5 or E18.5 (Fig. 5F and not shown).

### Expression of key regulators of chondrocyte proliferation and endochondral ossification in the absence of *c-Myc*

**Figure 6:**
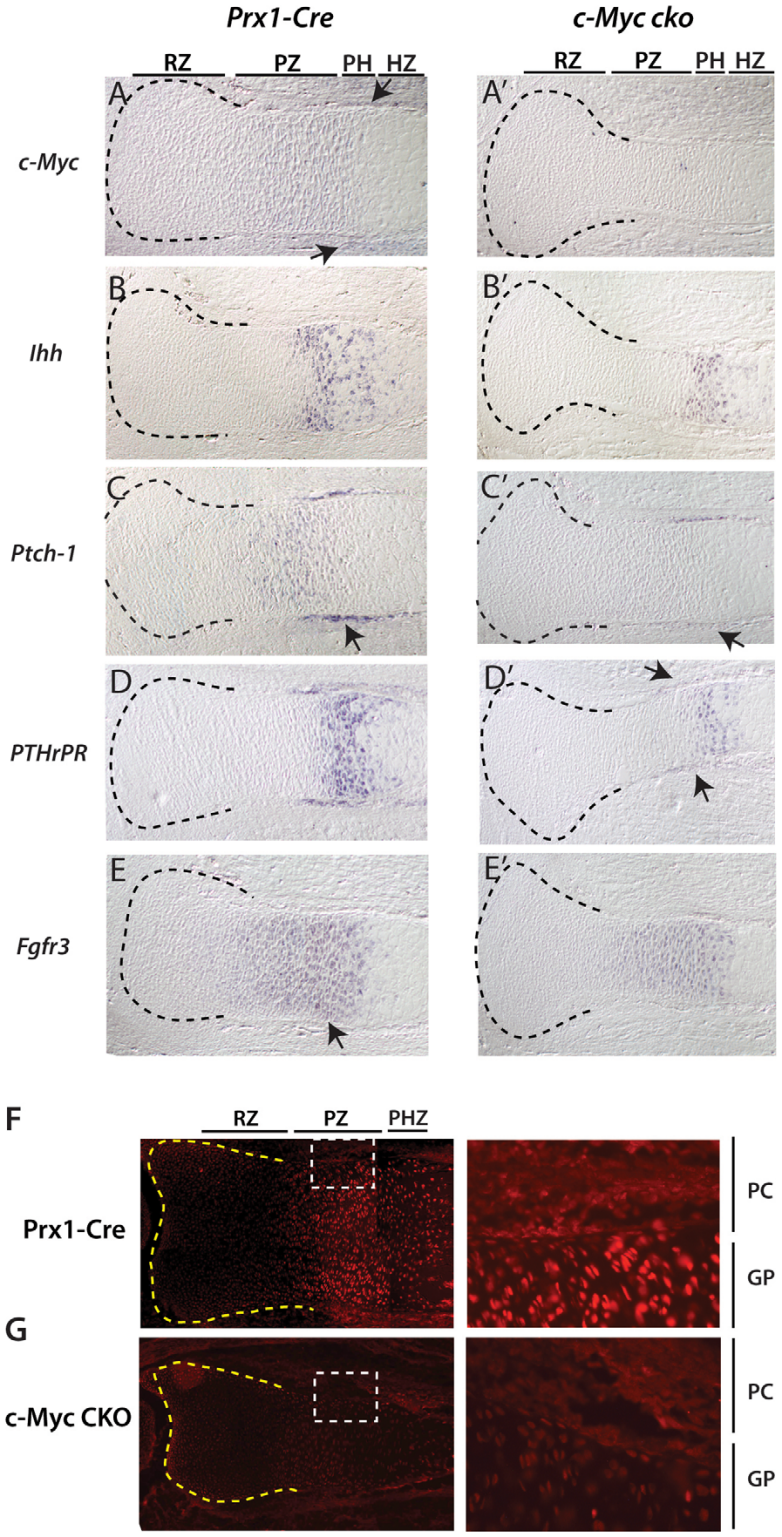
Decreased expression of key regulators of the transition from proliferation to prehypertrophy in chondrocytes in the absence of *c-Myc* deletion. (A–E') In situ hybridization (purple signal) of *c-Myc*, *Fgfr3*, *IHH*, Ptch-1 and *PTHrPR* in control and *c-Myc* proximal tibia at E16.5. Arrows point to perichondrial expression of *c-Myc, Ptch-1* and *PTHrPR* expression. Approximate subregions of the growth plate are indicated. (F, G) pRpCTD (pCTD) immunohistochemistry of control and Prx1-Cre c-Myc mutant tibias at E16.5. Higher magnification views are shown at right. Approximate subregions (RZ, PZ and PHZ) of the growth plate are indicated and perichonium (Pc) and growth plate (GP) regions of the higher magnification are indicated.

The reduced number of chondrocytes in proliferative and hypertrophic zones of *c-Myc* deficient growth plates, along with reduced numbers of *Col1*-, *osteocalcin*- and *Runx2*-positive osteoblasts in the perichondrium, suggested that loss of *c-Myc* might broadly influence signaling systems that promote proliferation of chondrocytes and osteoblasts. Consistent with this idea, both the expression domains and expression intensity of *IHH*, *Ptch-1* and *PTHrPR* were markedly reduced in the absence of c-Myc (Fig. 6A-D'). In addition, expression of *Fgfr3*, which is expressed primarily in the proliferative zone but is a negative regulator of chondrocyte proliferation [35], was also reduced (Fig. 6E,E'). Thus, like *Runx2*, *Sox9* and *Vegfa*, each of these genes was expressed in the appropriate location within the growth plate and perichondrium (where applicable), but their expression levels appeared reduced.

Although the above results raised the possibility that a general reduction in gene transcription might be an important mechanism underlying the effect of c-Myc deficiency on skeletogenesis, it was unclear how much the observed reduction in mRNA signals was related to decreased number/density of chondrocytes and osteoblasts. To address this issue we examined expression of Serine 5 phosphorylation of the C-terminal tail (pCTD) of RNA Polymerase II, an event required for transcription to be initiated [36]. Interestingly, pCTD was found to be robust in the proliferative compartment of the growth plate and appeared to closely match the *c-Myc* expression domain (Fig. 6F). Moreover, pCTD was remarkably low in the equivalent compartment of growth plates lacking *c-Myc* (Fig. 6G). Although less robust in perichondrium relative to the proliferative zone of the growth plate of control *Prx1-Cre* mice, pCTD was also clearly reduced in *c-Myc* deficient perichondrium (Fig. 6F, G). These results support previous data suggesting that loss of c-Myc affects global transcription in vivo [36], [24], and suggest that suppression of global transcription, and not altered transcriptional regulation of any specific set of Myc target genes, may be ultimately responsible for the effects of c-Myc loss on endochondral ossification and bone size.

### Combined loss of *N-Myc* and *c-Myc* in limb bud mesenchyme causes defects in limb bud outgrowth that can be attributed to deletion of *N-Myc*

**Figure 7:**
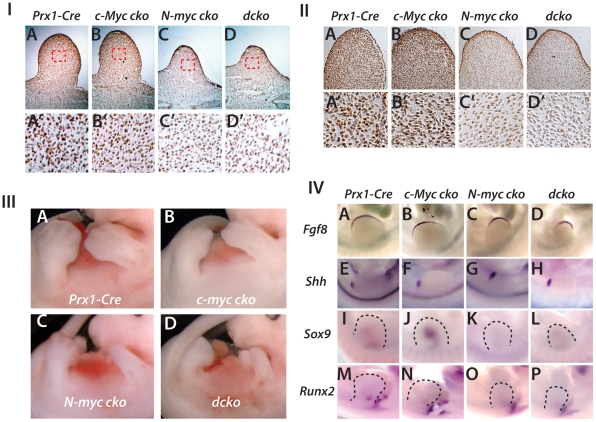
Combined *c-Myc* and *N-Myc* deletion mimics deletion of *N-Myc* alone in the early limb bud and prevents Sox9 upregulation caused by *c-Myc* deletion. (IA–D) BrdU labeling of limb buds at E10.5 for the indicated genotypes. Higher magnification views of boxed areas (red) are shown in A'–D'. Similar results were observed in sections from three different limb buds. (IIA–D') pRpCTD immunohistochemistry of E10.5 limb buds of the indicated genotypes with high (IIIA–D) Comparison of limb size and morphology for the indicated strains at E12.5. (IV) Whole mount in situ hybridization for *Fgf8*, *Shh* and *Sox9* at E10.5 and *Runx2* at E11.5 in *Prx1-Cre*, *c-Myc cko, N-Myc cko* and dcko embryos.

N-Myc supports proliferation of undifferentiated mesenchymal cells in the limb bud and therefore contributes to the generation of *Sox9*-positive cells of the limb bud core that form the precartilaginous condensation [25], [3]. In contrast to *N-Myc* deletion, which consistent to previous results caused a strong decrease in BrdU incorporation in limb bud mesenchyme (but not in ectoderm) at E10.5, *Prx1-Cre* deletion of *c-Myc* had little or no effect on mesenchyme proliferation at this stage (Fig. 7IA–C, also see Fig. 1H). These effects on proliferation are consistent with the different expression patterns of *N-Myc* and *c-Myc* in the early limb bud (Fig. 1A–D, [25], [3]. Similar to these differential effects on BrdU incorporation, pCTD was strongly diminished in the mesenchyme of *N-Myc* and *dcko* mutants, but not in *c-Myc* mutants (Fig. 7IIA–D'). Note that pCTD levels in the ectoderm serves as a control because Prx1-Cre is not active in the ectoderm.

Whereas deletion of *N-Myc* by *Prx1-Cre* caused formation of small misshapen limb buds and subsequently small skeletal elements and syndactyly [25], loss of *c-Myc* had little or no consistent effect on limb bud size and shape (Fig. 7IB, IIIB), but affected the initial expression of *Sox9* and *Runx2* at limb bud stages (Fig. 2A', 7IVJ, N) and resulted in small skeletal elements as described above. In contrast, *N-Myc* deletion suppressed the initial *Sox9* and *Runx2* expression at E10.5 and E11.5 respectively (Fig. 7IVC, N) and more severely affected limb bud development, causing smaller and narrower forelimb and hindlimb buds (Fig. 7IIIC and [25]. *N-Myc* deficient forelimb buds were more severely affected than hindlimb buds (Fig. 7IIIC), a result in line with the more robust *Prx1-Cre* activity in the emerging forelimb bud than in the hindlimb bud [31]. Consistent with *N-Myc* regulating early undifferentiated limb bud mesenchyme and limb bud outgrowth and *c-Myc* acting primarily at later stages, combined deletion of *N-Myc* and *c-Myc* with *Prx1-Cre* (*dcko*), like deletion of *N-Myc* alone, resulted in strongly reduced cell proliferation and produced small limb buds at E10.5 (Fig. 7IC, D). Similarly, *N-Myc* deficient limb buds at E12.5 exhibited the characteristic shape and reduced size of *dcko* limb buds, with forelimb buds being more severely affected (Fig. 7IIIC, D and data not shown).

The small size of *dcko* limb buds was not due to any substantial defects in expression of *Fgf8* in the apical ectodermal ridge or *Sonic hedgehog* (*Shh*) in the zone of polarizing activity (Fig. 7IVA–H). Thus, defects in limb bud growth of *dcko* mice occurred downstream or parallel to the activities of the major limb bud signaling centers regulating proximal-distal and anterior-posterior limb development. The upregulation of *Sox9* at E10.5 caused by *c-Myc* deletion alone did not occur when *N-Myc* was also deleted or in *dcko* mutants (Fig. 7IVK, L and also see Fig. 2B'), and the initial *Runx2* expression domain in the limb bud was reduced in *N-Myc* and *dcko* mutant limb buds at E11.5 Fig. 7IVO, P). These results are consistent with a requirement for *N-Myc* in the efficient production of cells that will become *Sox9* and *Runx2* expressing osteo-chondro progenitors prior to *c-Myc* expression in these cells or their derivatives.

### Combined loss of *N-Myc* and *c-Myc* in limb bud mesenchyme causes severe skeletal hypoplasia and loss of proximal elements

**Figure 8:**
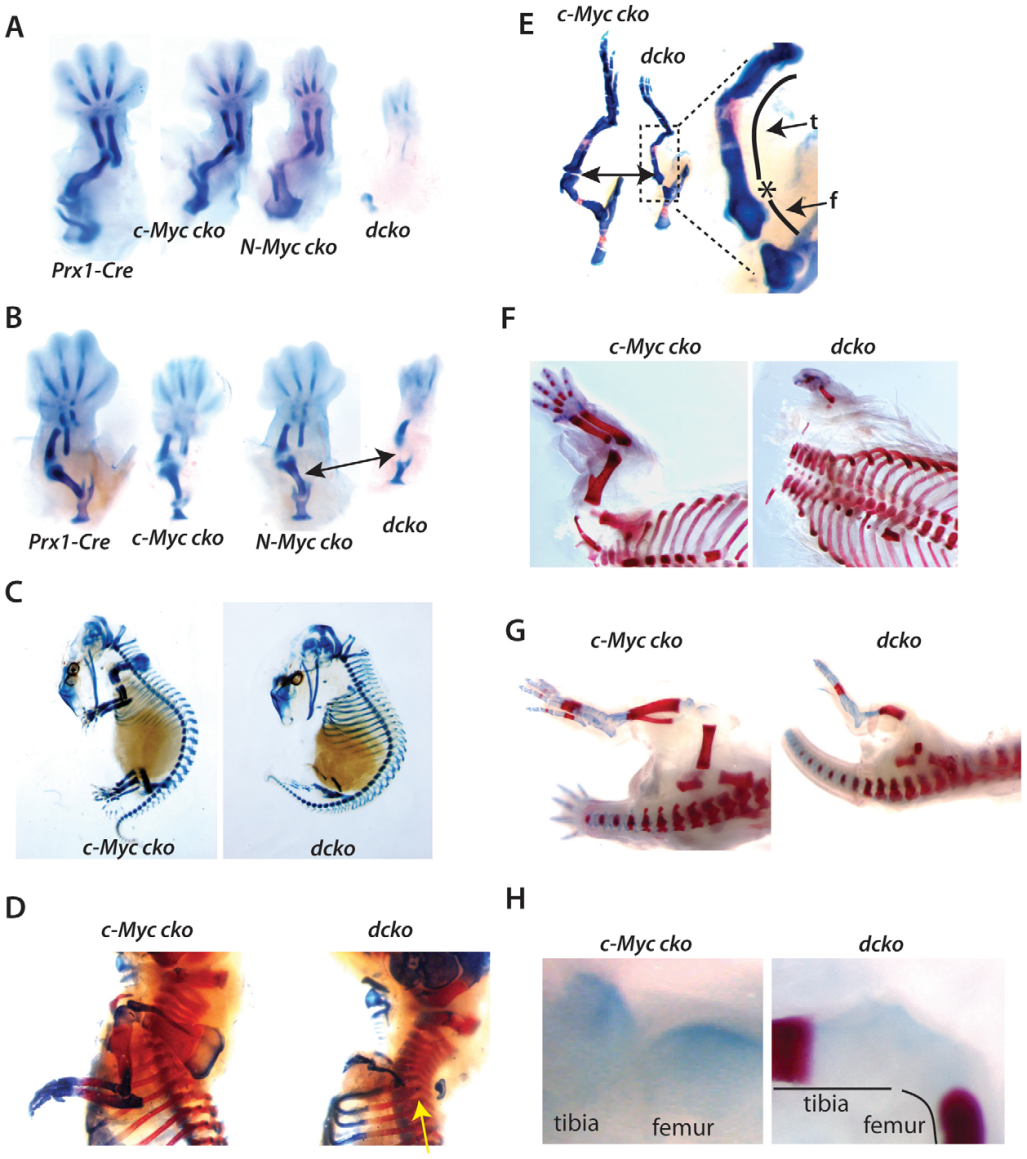
Combined c-Myc and N-Myc deletion results in severe limb skeletal agenesis. (A, B) Alcian blue stained forelimbs respectively of the indicated mouse strains at E13.5. (C) Alcian blue stained *c-Myc* and *dcko* mutants at E15.5. (D) Alcian blue and Alizarin red stained preparation of E18.5 embryos showing forelimbs (yellow arrow points to the absence of humerus and distal scapula). (E) E18.5 hindlimb region with higher magnification image at right showing fusion of the femur (f)-tibia (t) joint (*) of *dcko* embryos. (F) Forelimbs of E20.5 embryos showing absence of humerus and scapula elements in *dcko* embryos. (G) Hindlimbs of *c-Myc* and *dcko* mutants at E20.5. (H) Higher magnification images of the femur-tibia joint region of the E20.5 *dcko* mutant shown in (G).

While *N-Myc* and *c-Myc* have unique and mostly complementary expression patterns in the developing limb, the initial forelimb cartilage templates of *Prx1-Cre c-Myc* and *N-Myc* mutants at E13.5 exhibited a similar reduction in overall size (Fig. 8A). The forelimb skeleton of dcko embryos at E13.5 revealed a minimally developed cartilage template with no evidence of a humerus element or proximal scapula tissue (Fig. 8A). The hindlimb elements of *N-Myc* mutants were not as severely affected as *c-Myc* mutants, a result consistent with delayed *Prx1-Cre* activity in hindlimb buds relative to forelimb buds (32, Z-QZ and PJH not shown) and the demonstrated role of N-Myc (but not c-Myc) in proliferation of limb bud mesenchyme (Fig. 7I). Furthermore, while the E13.5 hindlimb skeleton of *dcko* embryos was grossly hypoplastic and the femur, like the humerus in the forelimb was missing (Fig. 8B), they were less severely affected than *dcko* forelimbs (Fig. 8A), a result that again can be attributed to delayed deletion of *N-Myc* in hindlimb mesenchyme relative to forelimb. Moreover, PCR genotyping of E10.5 limb bud mesenchyme showed robust and essentially equivalent *Prx1-Cre* mediated deletion of *c-Myc* and *N-Myc* (data not shown), indicating that any differences observed were not due to differences in ablation efficiency. Taken together with our analysis of *Prx1-Cre* and *Sox9-Cre c-Myc* mutants, these results are consistent with N-Myc and c-Myc playing distinct and complementary roles during development of the limb skeleton.

The basic observations of *dcko* limb skeletons at E13.5 are reinforced by examination of the skeleton at later stages. At E15.5, very little can be deciphered from forlimbs of dcko embryos because they are so poorly developed (Fig. 8C). However, at E18.5 and E20.5, it was clear that forelimbs of *dcko* embryos lacked a radius, humerous, and proximal scapula tissue (Fig. 8D, F) and the hindlimb exhibited a hypoplastic skeleton with poor ossification as indicated by Alizarin red staining (Fig. 8E, G). In the *dcko* hindlimbs, the fibula was absent and the femur was more affected than the tibia (Fig. 8E, G). The hindlimb girdle was less affected than the scapula in *dcko* mice, with the latter being comprised of only the most proximal component (Fig. 8D). In addition, fusion between the femur and tibia in *dcko* hindlimbs was consistently observed (Fig. 8E, H and not shown). Finally, in the autopod of *dcko* forelimbs, only the central digits (typically digits 3 and 4) were formed, and like *N-Myc* mutants [25] they exhibited complete syndactyly with boney fusions (Fig. 8F and data not shown).

### Defects in endochondral growth and ossification caused by combined loss of *N-Myc* and *c-Myc*

**Figure 9:**
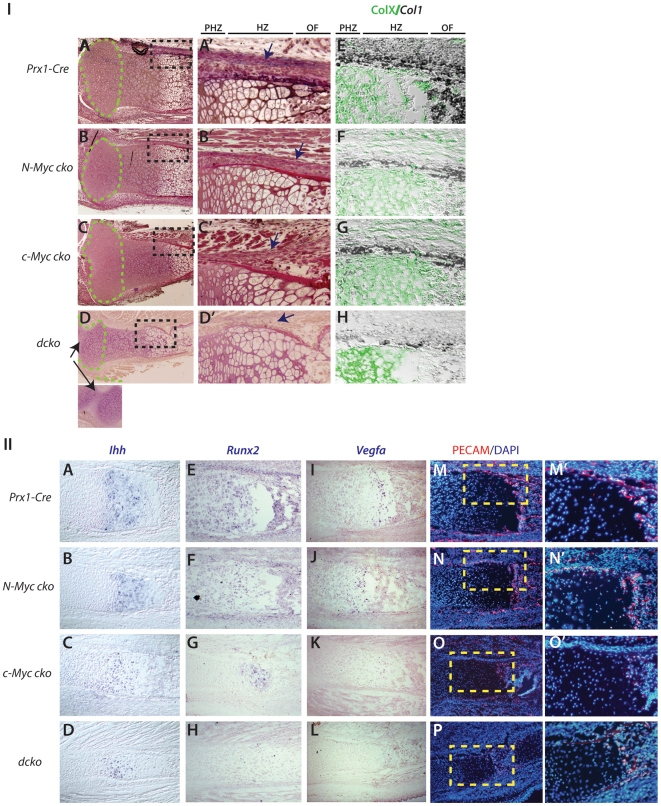
Unique and additive contributions of *N-Myc* and *c-Myc* to endochondral growth and ossification. (IA–D') Histological comparison of E18.8 proximal tibia of *N-Myc*, *c-Myc* and *dcko* mutant embryos. Green dashed lines outline the epiphyseal head regions and higher magnification views of the black-boxed regions are shown (A'–D'). Arrow in *dcko* image (D) indicates fusion between tibia/fibula and femur. (E–H) Combined ColXa1 immunohistochemistry (green) and *Col1* in situ hybridization (dark blue) at E18.5. (II) In situ hybridization of *Ihh* (A–D), *Runx2* (E–H) and *Vegfa* (I–L) and PECAM immunohistochemistry (M–P) in tibia sections at E18.5 from the indicated mouse strains. Yellow boxed regions in M-P are shown in M'–P'.

Both forelimb and hindlimb elements of *dcko* mice remained extremely small throughout development and exhibited very small or no primary ossification centers, suggesting persistent disruption of endochondral growth and ossification. Because forelimb skeletal elements that formed in *dcko* mice were extremely small and misshapen, we examined endochondral growth and ossification on less severely affected hindlimb. Histological analysis of E18.5 proximal tibia sections from *dcko* mice revealed a combination of architectural features found in individual *c-Myc* and *N-Myc* cko mice (Fig. 9IA,B). First, the epiphyseal head, which encompasses the resting/progenitor zone of the growth plate, was consistently reduced in size in both *N-Myc* mutants and *dcko* mice, but not in *c-Myc* mutants (Fig. 9IA–D – green dashed line). Second, the characteristic narrow and constricting growth plate from the proliferating zone to the ossification center of *c-Myc* mutants was present in *dcko* mice, but not in *N-Myc* mutants (Fig. 9IA–D'). While chondrocytes in the growth plates of individual *Myc dcko* mutants differentiated into *ColXa1*-positive hypertrophic cells, the reduced numbers of chondrocytes in the prehypertrophic/hypertrophic region, particularly in *c-Myc* mutant and *dcko* growth plates, corresponded to markedly reduced levels of *Ihh, Runx2, Vegfa* (Fig. 9IIA–L). Moreover, decreased vascularization of the perichondrium and ossification front correlated with reduced *Vegfa* levels in *c-Myc* and *dcko* mutants. (Fig. 9IIM–O). As in *c-Myc* mutants (Fig.6B), these effects corresponded to severely reduced pRpCTD in *dcko* growth plate and perichondrium (Fig. S3). Suppressed pRpCTD at these locations in the hindlimb tissues analysed is primarily attributed to loss of c-Myc since *c-Myc* and not *N-Myc* is expressed at these sites and relative to *Prx1-Cre* deletion of *N-Myc* in the forelimb (early limb bud deletion*), Prx1-Cre* deletion of *N-Myc* in the hindlimb (late limb bud deletion) results in a less hypoplastic skeleton.

Finally, whereas *N-Myc* deficiency alone decreased the size of the epiphyseal head of the tibia (and other limb elements – not shown), and resulted in small declines in expression of *Ihh*, *Runx2*, *Vegfa* in the growth plate, it had a relatively strong effect on *Col1* and *Runx2* expression in the perichondrium, and on the thickness of perichondrium (Fig. 9IB, B', F, 9IIF). *c-Myc* deficiency also resulted in a thin perichondrium and reduced perichondrial expression of *Col1* and *Runx2* (Fig. 9IC, C', F, 9IIG). Consistent with both c-Myc and N-Myc contributing to osteoblast development, *Col1* and *Runx2* were further reduced in *dcko* mutants, which had very little identifiable perichondrial tissue (Fig. 9ID, D', 9IIH,L).

## Discussion

**Figure 10:**
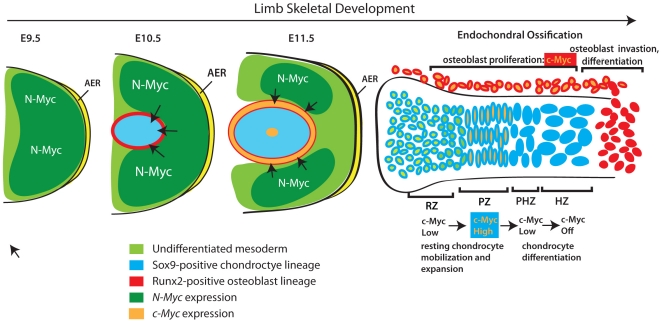
Model for the combined actions of N-Myc and c-Myc during limb development. *N-Myc* expression in the early limb bud promotes expansion of undifferentiated multipotent mesenchymal cells. As the limb bud expands, secreted factors from the ectoderm (e.g. FGF and Wnt family members) that promote *N-Myc* expression and proliferation in underlying mesenchyme can no longer reach the most central cells and these cells exit the cell cycle and are fated to become Sox9-positive chondro-osteoprogenitors [3]. In addition to cell cycle exit, the hypoxic environment and *Hif1α* upregulation [44] appears to participate in fate determination steps that initiate the *Sox9*-positive chondrocyte and *Runx2*-positive osteoblast lineages. The commencement of low-level *c-Myc* expression in these lineages is proposed to promote their proliferative expansion, but maintain their multipotent progenitor character. Subsequent elaboration of the growth plate in cartilage anlagen leads to the regional partitioning of *c-Myc* expression where it acts as part of a program to mobilize resting chondrocytes to proliferate and commit to the chondrocyte terminal differentiation program. Although c-Myc expression in perichondrial osteoblasts likely contributes to their proliferation, by controlling the number of hypertrophic chondrocytes produced, *c-Myc* impinges on the production of critica l factors produced from these cells that also promotes osteoblast development and endochondral ossification.

In this study we show that c-Myc and N-Myc function cooperatively to drive limb development. Together with previous studies on the role of N-Myc in limb development [25], [3], our data support a model in which N-Myc plays an important role in limb bud outgrowth and production of mesenchymal cells that give rise to chondrocyte and osteoblast progenitors, while c-Myc participates in the proliferative expansion of these latter cells (Fig. 10). Moreover, c-Myc appears to be particularly important in the growth plate, where it controls bone size by regulating the transition of resting chondrocytes to proliferating chondrocytes and therefore the ultimate number of cells that become mature chondrocytes.

### c-Myc regulation of endochondral growth and ossification

In many respects, the dynamics of growth plate function and endochondral ossification resembles that of other homeostatic tissues such as skin and gut epithelium and the hematopoetic system [37]. In these latter settings, c-Myc and/or N-Myc play prominent roles in regulating the maintenance and expansion of resident stem cells as well as their commitment towards more differentiated cell types. This is probably most apparent in the skin where c-Myc upregulation is associated with the transition from slow-dividing keratinocyte stem cells to highly proliferative transit-amplifier cells, whose multiplication is needed to meet the requirements of an organ that is continuously sloughing off terminally differentiated cells [38]. While endochondral bone growth is finite, the role of c-Myc in producing differentiated chondrocytes during this process appears to provide an analogous function. After the initial formation of cartilage, c-Myc promotes endochondral bone growth by stimulating largely quiescent chondrocytes within the reserve zone to proliferate in conjunction with their commitment to the chondrocyte differentiation program. As a result, c-Myc appears to alter the architecture of the growth plate and control endochondral ossification and the ultimate size of bones by regulating the number of terminally differentiated chondrocytes produced. While the detailed mechanism by which c-Myc regulates chondrocyte proliferation remains unclear, we show that transcription initiation by RNA PolII, as marked by phosphorylation of its CTD, is strongly upregulated in proliferating chondrocytes where *c-Myc* is expressed (Fig. 6B, Fig. S3). Moreover, the failure of pCTD to be upregulated and an apparent reduction in expression of various important regulators of growth plate function in the absence of c-Myc is consistent with the notion that Myc broadly affects the transcriptional state of cells and that a Myc-induced hypertranscriptional state is associated with, and perhaps necessary for, stimulating and maintaining high levels of cell proliferation [36]. Moreover, the striking reduction in pCTD in limb bud mesenchyme of *Prx1-Cre N-Myc* mutants suggests that N-Myc and c-Myc have the same general effect on global gene transcription.

While c-Myc promotes chondrocyte proliferation in the growth plate, our data indicate that it also influences proliferation in the perichondrium and is required for efficient production of osteoblasts. *c-Myc* is expressed in a ring pattern around the core of the limb bud at E11.5 and in perichondrium of cartilage anlagen at E13.5 (Fig. 1D), locations where osteoblast progenitors are thought to first arise and expand in number respectively [5], [21]. Moreover, perichondrial *c-Myc* expression at E16.5 appears to overlap with expression of *Ptch-1* and *Pthr1* (Fig. 6A), which are well-established regulators of osteoblast proliferation [*7]*, [8**]. However the finding that *Osx1-Cre* deletion of *c-Myc* resulted in no discernable skeletal phenotype (Fig. S2 and data not shown), suggests that any cell autonomous role of *c-Myc* in osteoblast proliferation and behavior must take place prior to *Osx1*-related activities that are involved in osteoblast differentiation [8]. Indeed, the finding that *c-Myc* is upregulated and proliferation is induced in osteoblasts upon deletion of *Osx1* [39] supports the idea that Osx1 functions at post-mitotic stages in the osteoblast differentiation program at least in part by suppressing c-Myc expression.

Although our results implicate c-Myc in the control of osteoblast development, a potential cell non-autonomous role for *c-Myc* in regulating osteoblast proliferation and behavior may be linked to its control of chondrocyte proliferation and the number of differentiated chondrocytes produced in the growth plate. This is because the transition from proliferating chondrocytes to prehypertrophic chondrocytes coincides with expression of factors such as *Runx2*, *Vegfa*, and *Ihh*, whose activities help coordinate chondrocyte proliferation and differentiation during endochondral ossification [8]. Whereas Ihh influences chondrocyte proliferation and osteoblast proliferation and/or survival through binding its receptor Ptch-1 in the growth plate and perichondrium respectively, Vegfa stimulates vascularization of the cartilage template and hence migration of osteoblasts to the ossification front [8]. Since Ihh plays an important role in maintaining chondrocyte proliferation, the reduced number of *Ihh* producing prehypertrophic chondrocytes in *c-Myc* mutants and the apparent reduction in *Ihh* expression in those cells (Fig. 6C, C' and Fig. 8B, C) is predicted to further diminish both chondrocyte and osteoblast proliferation. Indeed, it is possible that Ihh requires *c-Myc* to promote or maintain chondrocyte proliferation and the production of an appropriate number of prehypertrophic chondrocytes expressing *Ihh*, as well as *Runx2*, *Vegfa* and other factors that play critical roles in endochondral growth and ossification. The production of fewer prehypertrophic chondrocytes in the absence of *c-Myc* may therefore be responsible for a cascade of secondary events that both further reduce the number of chondrocytes and osteoblasts in developing bones and diminish the ability of those produced to participate in endochondral ossification.

### Coordination of limb skeletal development through sequential N-Myc and c-Myc expression

The role of N-Myc in limb skeletal development appears to primarily be involved in promoting the expansion of undifferentiated mesenchymal cells in the limb bud that will contribute to formation of the precartilaginous condensation from which chondro-osteoprogenitors develop [25], [3]. Results shown here suggest that the activities are related to global effects on initiation of gene transcription (Fig. 7IIC,D). However, N-Myc may not have a uniform effect on condensation formation or chondro-osteoprogenitors since its deletion has a preferential effect on proximal elements, which was particularly evident in the *dcko* mice (Fig. 7V, VI, Fig. 8). Interestingly, *N-Myc* expression is fairly uniform in distal limb bud mesenchyme as the limb bud emerges, but then develops a strong lateral bias around E11.5 [25]. Thus, the relatively strong affect *N-Myc* deficiency has on the development of proximal elements may reflect a role for *N-Myc* in sustaining the production of cells that preferentially contribute to the prospective proximal region of the nascent condensation. Since retinoic acid signaling controls formation of the proximal limb skeletal elements [2], N-Myc may be involved in either regulating or mediating retinoic signaling in this setting. Further characterization of proximal limb development and segmentation in *N-Myc* and *dcko* mutants may help illuminate a potential role for N-Myc in retinoic acid signaling and how undifferentiated mesenchyme contributes to the regional specification and growth of the developing limb skeleton.

Genetic swap experiments indicate that *N-Myc* and *c-Myc* are functionally redundant during development [40], raising the question of why there is a transition from *N-Myc* expression in undifferentiated limb bud mesenchyme to *c-Myc* expression in chondrocyte and osteoblast lineages? In the developing limb, this transition coincides with a shift from highly proliferative undifferentiated mesenchyme that strongly express *N-Myc*, to the largely non-proliferative precartilaginous condensation at the core of the limb bud where *N-Myc* is no longer expressed [25], [3] and where *c-Myc* expression appears to surround (Fig. 1B, H). Therefore, one possibility is that the absence of both N-Myc and c-Myc proteins in the central core of the limb bud and a concomitant exit from the cell cycle of its residents serves as part of a mechanism that leads to the development of chondrocyte and osteoblast lineages. Consistent with this idea, preliminary data from mice in which ectopic *c-Myc* expression was targeted specifically to *Sox9*-expressing cells of the precartilaginous condensation show poor formation of the early cartilage template and defects in endochondral ossification that leads to short skeletal elements (Z-Q.Z, C-Y.S and P.J.H., unpublished). Thus, given the well-established role of Myc in promoting and maintaining the pluripotent or multipotent character of stem and progenitor populations and the ability of forced Myc expression to suppress cell fate transitions and cell differentiation [24], [23], [41], a transient period of low/no Myc expression and associated cell cycle exit may facilitate epigenetic reprogramming events and related changes in gene expression required for promoting and/or fixing new cell fates, including expression of Sox9 and development of chondro-osteoprogenitors in condensing mesenchyme of the limb bud. Interestingly, even though either *N-Myc* or *c-Myc* are expressed in most if not all proliferating cell types [42], [27], they are rarely expressed in the same cells and switching from expression of one *Myc* gene to another appears to be a common event associated with various developmental transitions during organogenesis and in homeostatic tissues [37]. Thus, while providing essentially the same activity, the unique (but potentially partially overlapping) transcriptional codes of *N-Myc* and *c-Myc* may have evolved to facilitate and coordinate developmental transitions that incorporate, and presumably require, a transient exit from the cell cycle. Understanding these codes may provide insight not only into the underlying mechanisms that drive various critical developmental transitions, but also into how to manipulate the expression of Myc proteins in vivo to facilitate the therapeutic proliferative expansion of specific progenitor populations in injury and disease settings.

## Materials and Methods

### Mice

*c-Myc*-floxed mice [30] were mated with *Prx1-Cre* mice (Logan et al. 2002), *Osx1 -Cre* mice [33] and *Sox9-Cre* [5] on a mixed background of C57BL/6 and 129. *c-Myc*-floxed mice were mated with *N-Myc*-floxed mice [43] and *Prx1-Cre* mice to generate double conditional knockout mice. All mice were maintained and used in accordance with animal protocols approved by the Oregon Health & Science University Institutional Animal Care and Use Committee.

### Histological analysis

Alcian blue, Alizarin red staining and von Kossa staining were performed using standard procedures. To examine detailed bone architecture (Fig. 8A–D), limbs were fixed with 1.5% glutaraldehyde/1.5 paraformaldehyde in DMEM w/0.6% Ruthenium hexamine trichloride overnight at 4 degrees followed by 3 changes of DMEM containing 0.6% RHT over 15 minutes. Tibias were dissected out, osmicated for 90 min in 1% OsO4 w/0.6% RHT in DMEM, rinsed in DMEM, dehydrated from 30 to 100% EtOH, rinsed in propylene oxide and infiltrated in Spurr's epoxy (microwave assisted). Samples were cut and stained using the epoxy tissue stain from EMS (Charleston SC).

### Immunohistochemistry and in situ hybridization

Embryos were fixed in 4% paraformaldehyde at 4°C overnight and processed for whole mount in situ hybridization using digoxigenin-UTP-labeled probes or embedded in OTC and sectioned before hybridization as previously described (Brent et al. 2003). For immunohistochemistry, limb sections were incubated with the following primary antibodies: anti-phospho-Rpb1 CTD - Cell Signaling, Danvers, MA, anti-CD31(PECAM) - BD Bioscience, San Jose, CA, Type X collagen - Dr. William Horton), followed by incubations with Alexa Flour-conjugated secondary antibodies (Invitrogen, Carlsbad, CA) or DAB staining using the Vectastain ABC Kit (Vector Laboratories, (Burlingame CA) as previously described [25].

### BrdU incorporation and TUNEL assays

BrdU (Sigma) labeling (1.5 hr) was performed using BD Biosciences (San Jose, CA) BrdU In Situ Detection Kit as previously described [25]. Anti-BrdU stained sections were counterstained with Hematoxylin. TUNEL was performed on fixed sections using the Roche (Basal, Switzerland) In Situ Cell Death Detection Kit. The percentage of BrdU positive cells was determined from counts of total cell numbers within comparable regions of sectioned limb buds or proximal tibia. Counts were determined from at least three serial sections from two or more independent experiments. Data are presented as mean ± SEM. Statistical significance of differences was calculated using Student's *t*-test.

### Cell density measurements

Cell density measurements were made from within equivalent areas of proliferative and hypertrophic zones. The specific regions selected were identified by their characteristic cell morphology, as well as by BrdU labeling of serial sections and ColX staining. DAPI stained sections from at least five different tibias were used in these calculations. Data are presented as mean ± SEM and statistical significance of differences were calculated using Student's *t*-test.

## Supporting Information

Figure S1 **Limb skeletal phenotype of mice with** ***Sox9-Cre*** **deletion of** ***c-Myc*** **.** (A) Alcian blue/Alizarin red staining of forelimb skeletal elements at E15.5 and (B) Alcian blue and H&E staining of E15.5 proximal tibia sections. Comparison of Alcian blue/Alizarin red staining of forelimb skeletal elements at E18.5 of the indicated mouse strains is shown.
(TIFF)

Figure S2 ***Osx1-Cre*** **deletion of** ***c-Myc*** **has little or no effect on endochondral ossification and growth.** (A) Alcian blue and Alizarin red skeletal preparations of the indicated mice at E18.5 mice. (B) Isolated hindlimbs E18.5 mice. (C) Alcian blue and H&E staining of proximal tibia sections.
(TIFF)

Figure S3 **Immunohistochemical analysis of RNA Polymerase CTD phosphorylation in E18.5 tibias of the indicated mouse strains.** Approximate locations of the Resting (RZ), Proliferative (PZ), Prehypertrophic (PHZ) and Hypertrophic Zones (HZ) are shown. Higher magnification images of the boxed regions are shown on the right.
(TIFF)
