# Supplementary figures and images for: Sequential and Coordinated Actions of c-Myc and N-Myc Control Appendicular Skeletal Development
Source: PLoS One. 2011 Apr 11;6(4):e18795. doi: 10.1371/journal.pone.0018795 (PMC3073980; doi:10.1371/journal.pone.0018795)

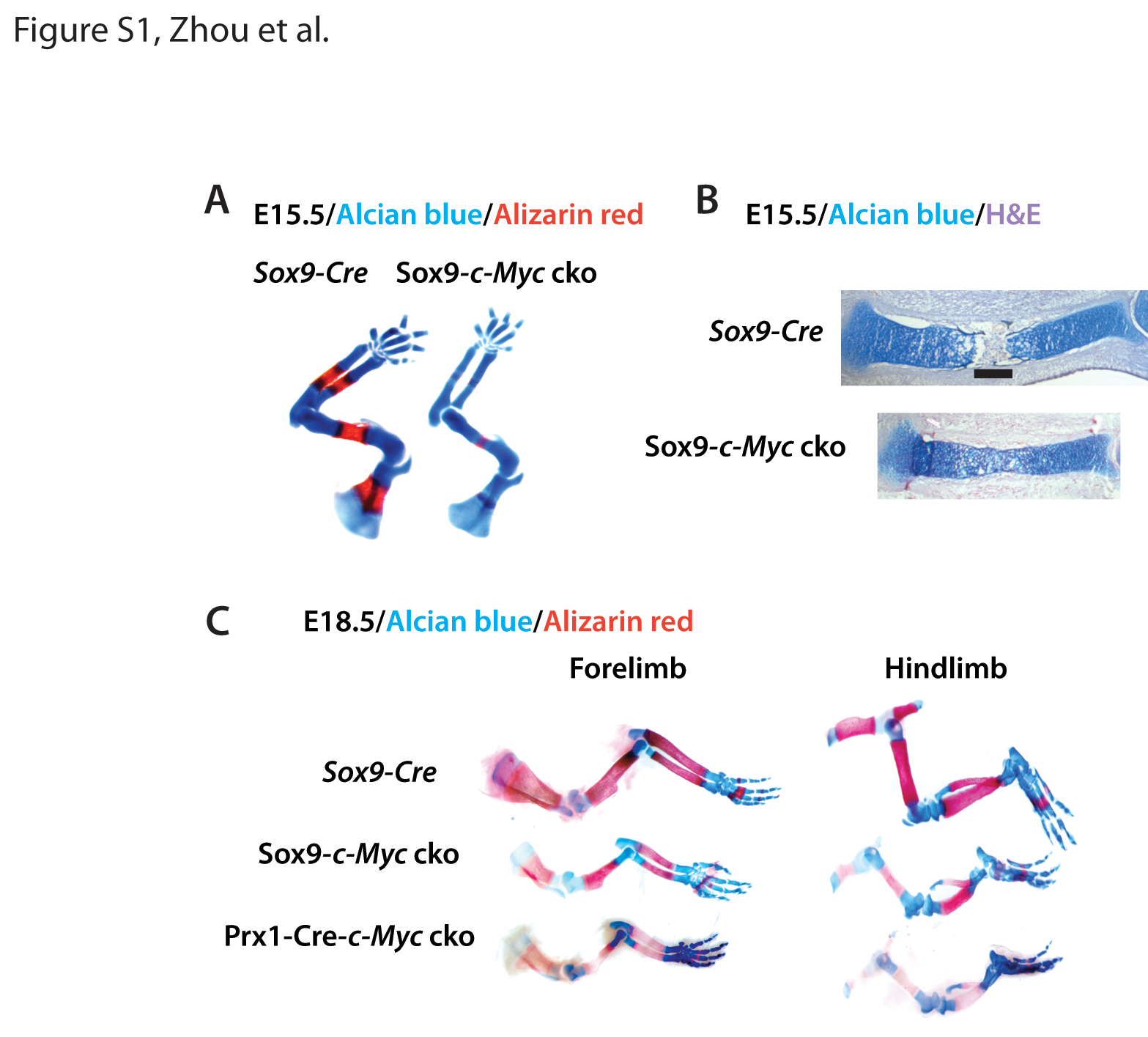

Supplement: Figure S1 — Limb skeletal phenotype of mice with Sox9-Cre deletion of c-Myc . (A) Alcian blue/Alizarin red staining of forelimb skeletal elements at E15.5 and (B) Alcian blue and H&E staining of E15.5 proximal tibia sections. Comparison of Alcian blue/Alizarin red staining of forelimb skeletal elements at E18.5 of the indicated mouse strains is shown. (TIFF) [file pone.0018795.s001.tiff]

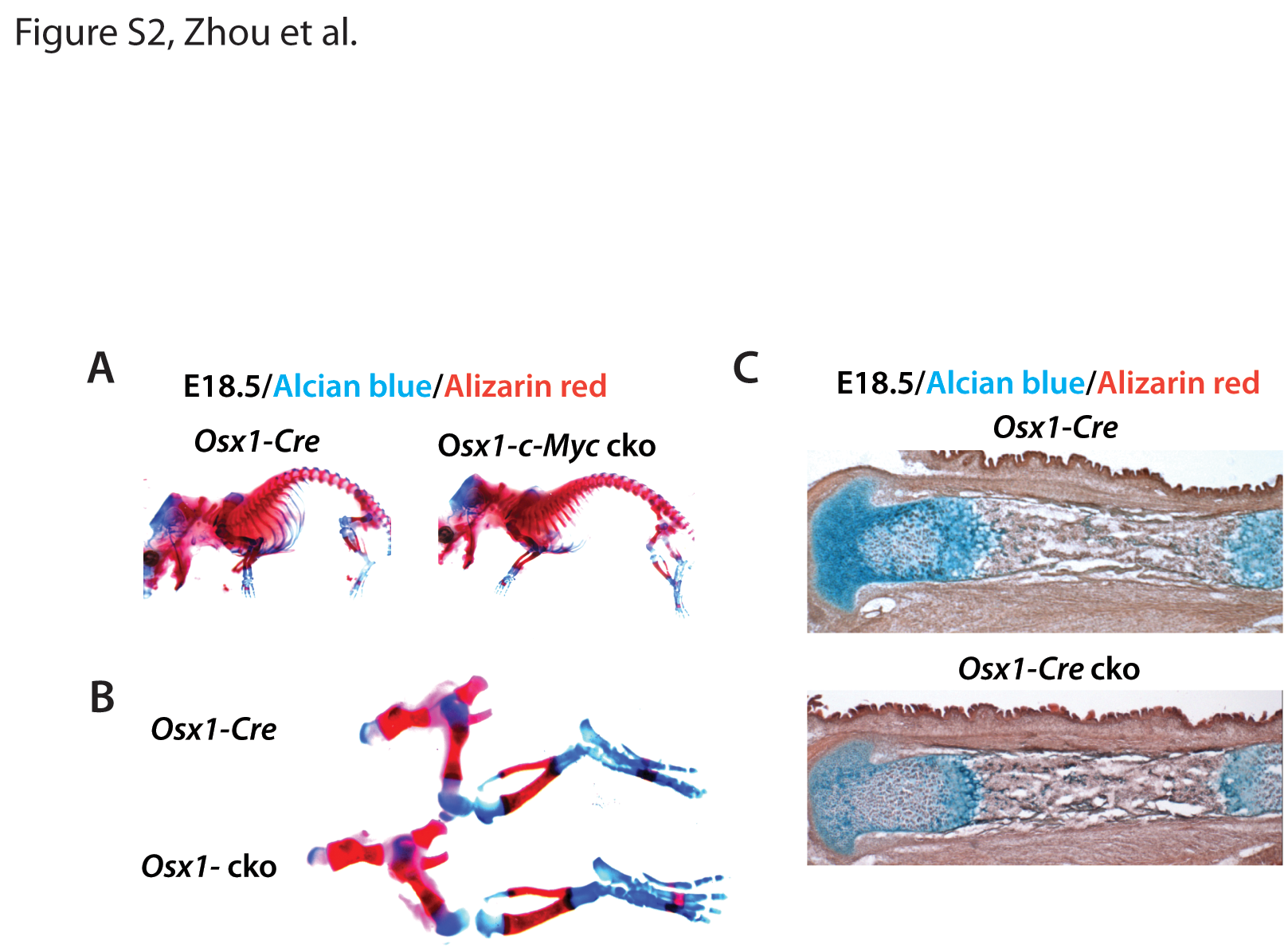

Supplement: Figure S2 — Osx1-Cre deletion of c-Myc has little or no effect on endochondral ossification and growth. (A) Alcian blue and Alizarin red skeletal preparations of the indicated mice at E18.5 mice. (B) Isolated hindlimbs E18.5 mice. (C) Alcian blue and H&E staining of proximal tibia sections. (TIFF) [file pone.0018795.s002.tiff]

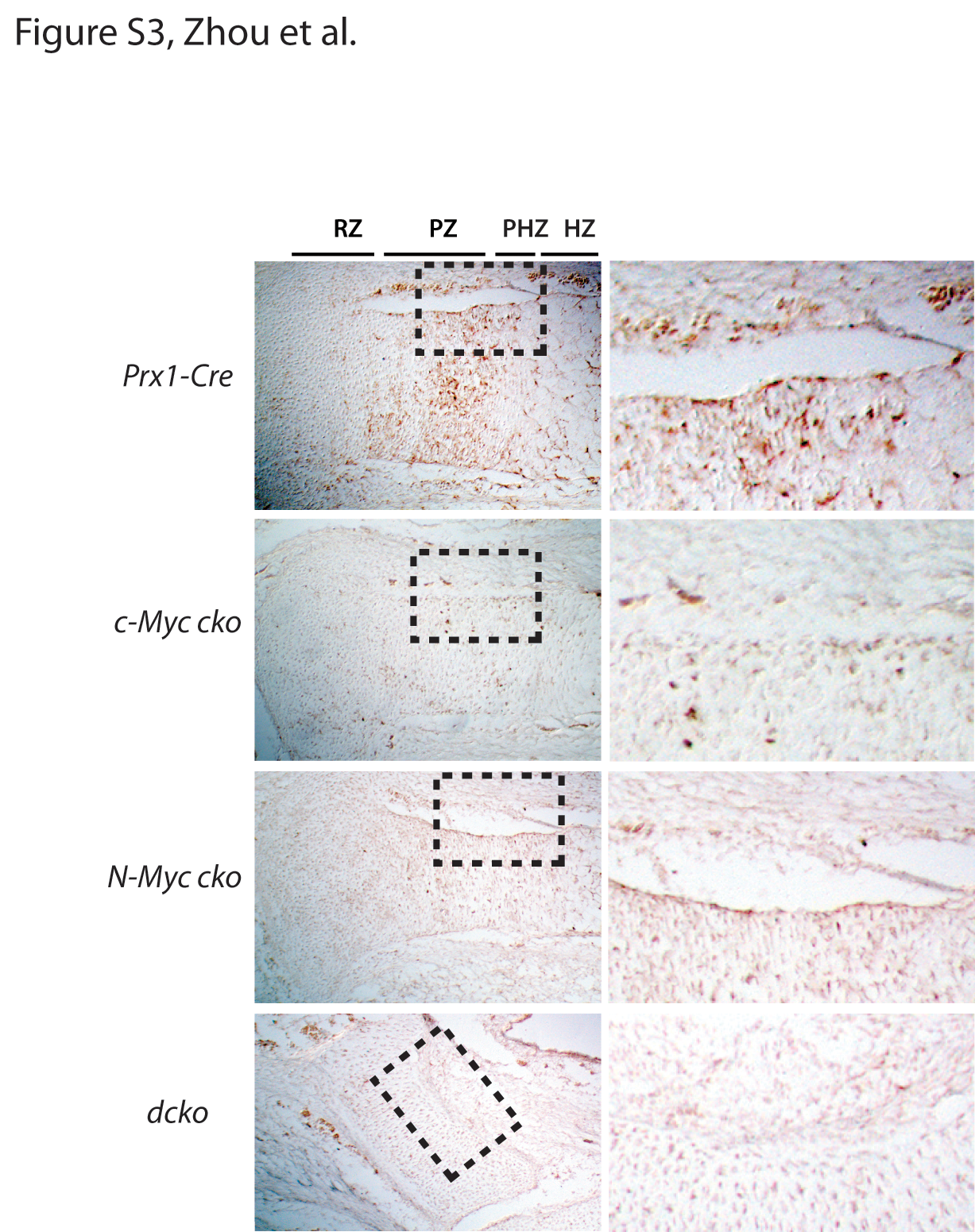

Supplement: Figure S3 — Immunohistochemical analysis of RNA Polymerase CTD phosphorylation in E18.5 tibias of the indicated mouse strains. Approximate locations of the Resting (RZ), Proliferative (PZ), Prehypertrophic (PHZ) and Hypertrophic Zones (HZ) are shown. Higher magnification images of the boxed regions are shown on the right. (TIFF) [file pone.0018795.s003.tiff]
